# GDF15/MIC1 and MMP9 Cerebrospinal Fluid Levels in Parkinson’s Disease and Lewy Body Dementia

**DOI:** 10.1371/journal.pone.0149349

**Published:** 2016-03-03

**Authors:** Walter Maetzler, Willy Deleersnijder, Valérie Hanssens, Alice Bernard, Kathrin Brockmann, Justus Marquetand, Isabel Wurster, Tim W. Rattay, Lorenzo Roncoroni, Eva Schaeffer, Stefanie Lerche, Anja Apel, Christian Deuschle, Daniela Berg

**Affiliations:** 1 Department of Neurodegeneration, Hertie Institute for Clinical Brain Research, Tuebingen, Germany; 2 German Center for Neurodegenerative Diseases (DZNE), University of Tuebingen, Tuebingen, Germany; 3 Fujirebio Europe NV, Technologiepark, Gent, Belgium; 4 Department of Epileptology, Hertie Institute for Clinical Brain Research, Tuebingen, Germany; Yale University School of Medicine, UNITED STATES

## Abstract

Based on animal and *ex-vivo* experiments, Growth/Differentiation Factor-15 (GDF15, also called Macrophage Inhibitory Cytokine-1, MIC1), a member of the transforming growth factor-beta family, and Matrix Metalloproteinase-9 (MMP9), a member of the matrix metalloprotease family may be potential markers for Lewy body disorders, i.e. Parkinson’s disease with (PDD) and without dementia (PDND) and Lewy body dementia (DLB). GDF15 has a prominent role in development, cell proliferation, differentiation, and repair, whereas MMP9 degrades, as a proteolytic enzyme, components of the extracellular matrix. In this study, cerebrospinal fluid GDF15 and MMP9 levels of 59 PDND, 17 PDD and 23 DLB patients, as well as of 95 controls were determined, and associated with demographic, clinical and biochemical parameters. Our analysis confirmed the already described association of GDF15 levels with age and gender. Corrected GDF15 levels were significantly higher in PDD than in PDND patients, and intermediate in DLB patients. Within Lewy body disorders, GDF15 levels correlated positively with age at onset of Parkinsonism and dementia, Hoehn & Yahr stage and cerebrospinal fluid t-Tau and p-Tau levels, and negatively with the Mini Mental State Examination. Remarkably, it does not relevantly correlate with disease duration. MMP9 was not relevantly associated with any of these parameters. Cerebrospinal GDF15, but not MMP9, may be a potential marker of and in Lewy body disorders.

## Introduction

At present, there is no biomarker in blood or cerebrospinal fluid (CSF) available that can differentiate between Lewy body disorders and controls with sufficient accuracy, not to mention the capability of such markers to predict the disease, or correlate with disease progression [1]. Growth/Differentiation Factor-15 (GDF15, identical to Macrophage Inhibitory Cytokine-1, MIC1) [2] and Matrix Metalloproteinase-9 (MMP9) belong to the Transforming growth factor-beta (TGF-beta) superfamily. Members of this family have prominent roles in development, cell proliferation, differentiation, and repair [3]. GDF15 is widely expressed in the central nervous system (CNS) and peripheral nervous system, most prominent in the choroid plexus, and secreted into the cerebrospinal fluid (CSF) [3]. The protein is up-regulated as part of the anti-inflammatory cytokine network within the CNS in response to injury and lesions [4,5], supplementing the roles of other members of the TGF-beta superfamily [6]. Moreover, GDF15 serves as an autocrine regulatory molecule in monocytoid cells, where expression of the protein may be required to limit or inhibit macrophage activation at a later phase [7]. Interestingly, GDF15 has been shown to act as a potent trophic and protective factor for dopaminergic neurons: According to recent results [3], GDF15 seems to be at least as potent as glial derived neurotrophic factor in promoting the survival of dopaminergic neurons, and normalizes motor behaviour. It also protects dopaminergic neurons against iron intoxication [8], induces neuronal survival by activating protein kinase B and glycogen synthase kinase via phosphatidylinositol 3 kinase, and attenuates formation of reactive oxidative species [9]. GDF15 probably acts directly on neurons [3]. Remarkably, GDF15 levels have been associated with cognitive performance and decline. Higher GDF15 levels were shown to be significantly associated with lower global cognition in a cohort of more than 1000 non-demented adults aged 70–90 years [10].

MMP9 is a stress response cytokine [11]. It is capable of degrading stable compounds of the extracellular matrix, as well as growth factors, cytokines, chemokines, cell surface receptors, and serine proteases inhibitors [12–15]. MMP9 is released from astrocytes, neurons and microglia as well as leukocytes and macrophages as an inactive proprotein, which is then activated by extracellular proteinases. MMP9 has been shown to be a potential biomarker for outcome prediction after neuron damage [14], and is particularly induced in neuronal lesions and cerebral ischemia [16]. Moreover, MMP9 might be important in remyelination processes as it facilitates oligodendrocyte regrowth [17]. MMP9 has been found to co-localize with neurofibrillary tangles, senile plaques and the vascular wall in post-mortem Alzheimer brains [18,19]. MMP9 synthesis is e.g. induced by Abeta_1-42_ peptide [20,21], and vice versa, MMP9 is capable of degrading Abeta_1-42_ [22].

Based on this knowledge about GDF15 and MMP9 in acute and chronic brain conditions with and without dopaminergic loss and neurodegeneration, we set out to measure CSF levels of these two proteins in a large cohort of Lewy body disorders, i.e. Parkinson’s disease without (PD no dementia, PDND), PD with dementia (PDD) and dementia with Lewy bodies (DLB), to determine their associations with demographical, clinical and routine CSF parameters.

## Methods

### Ethics Statement

The study was approved by the ethics committee of the Medical Faculty, University of Tuebingen (Germany), and was performed in accordance with the Declaration of Helsinki. All participants, or their next of kin in the case of cognitive impairment (MMSE score of 18 or below), provided their informed written consent.

### Study Participants

The study cohort consisted of 200 participants, i.e. 100 control persons and 100 patients with a Lewy body disorder. All samples were collected from donors who were either patients or study participants at the ward and outpatient clinic of the Neurodegenerative Department of the University of Tuebingen, Germany. From all included donors, information about gender, age, age at onset of Parkinsonism, age at onset of dementia, disease duration, Hoehn and Yahr scale (H&Y), Mini Mental State Examination (MMSE) scores and information about medication were collected. Diagnoses of PDND, PDD and DLB as well as of controls (exclusion of a neurological disease according to medical history and clinical examination) were made by movement disorders specialists according to established criteria [23–25]. One PDND patient and five controls were excluded from the final analysis due to missing data (neurodegenerative markers or MMP9 values not available), resulting in data available for final analysis of 99 patients with a Lewy body disorder (59 PDND, 17 PDD, and 23 DLB patients) and 95 controls. Excluded individuals did not relevantly differ from those included with regard to demographic and clinical data. Details are provided in **Table 1.**

**Table 1:** Demographical, clinical and biochemical data of the included cohorts.

		PDND	PDD	DLB	Controls	*p*-value
Individuals (f/m) [N]		59 (30/29)	17 (8/9)	23 (12/11)	95 (55/40)	0.76
Age [y]		67 (44–79)	75 (61–84)^§^	69 (50–82)	61 (38–79)^§^^#^^$^	<0.0001
Aao Parkinsonism [y]		62 (30–76)	62 (51–74)	67 (49–78)^§^	-	0.07
Duration of Parkinsonism [y]		4.0 (0.5–21.0)	10.0 (3.0–21.0)^§^	2 (0.5–13.0)^§^^#^	-	<0.0001
H&Y stage (1–5)		2 (1–4)	3 (2–4)^§^	2 (1–4)^#^	-	0.004
H&Y stage (1/2/3/4) [N]		9/40/7/3	0/7/6/4	5/8/8/2	-	0.0054
Aao dementia [y]		-	72 (58–79)	68 (49–79)^#^	-	0.04
Duration of dementia [y]		-	3.0 (0.2–5.0)	2 (0.5–12.0)	-	0.67
MMSE (0–30)		29 (23–30)	23 (14–26)^§^	20 (10–30)^§^	30 (25–30)^§^^#^^$^	<0.0001
LEDD [mg L-Dopa/day]		225 (0–1333)	600 (0–1016)^§^	150 (0–700)^#^	-	<0.0001
CSF Albumin [mg/L]		221 (127–391)	252 (109–440)	209 (96–427)	232 (83–575)	0.98
CSF Abeta_1-42_ [pg/mL]		678 (270–1458)	478 (213–771)^§^	530 (252–1241)^§^	825 (318–1446)^§^^#^^$^	0.0009^1^
CSF t-Tau [pg/mL]		176 (25–710)	225 (68–660)	279 (77–596)^§^	165 (32–481)^$^	0.15^1^
CSF p-Tau [pg/mL]		36 (14–111)	54 (12–109)^§^	50 (26–86)^§^	36 (13–76)^#^^$^	0.046^1^
CSF GDF15 [pg/mL]	All	200 (67–467)	292 (139–571)^§^	235 (101–453)^#^	184 (39–461)^#^	0.05^1^
	f	198 (67–467)	285 (139–473)	209 (101–356)^#^	169 (39–371)	0.13^1^
	m	202 (113–429)	296 (204–571)^§^	246 (185–453)	215 (78–461)	0.07^1^
CSF MMP9 [pg/mL]		50 (15–283)	47 (23–176)	42 (9–133)	46 (11–439)	0.40^1^

Aao, Age at onset; Abeta_1-42_, amyloid-beta_1-42_; CSF, cerebrospinal fluid; DLB, dementia with Lewy bodies; GDF15, growth/differentiation factor 15; f, female; H&Y, Hoehn & Yahr stage; LEDD, Levodopa equivalent daily dose; m, male; MMP9, matrixmetalloproteinase 9; MMSE, Mini Mental State Examination; PDD, Parkinson`s disease demented; PDND, Parkinson`s disease non-demented; p-Tau, phospho-Tau; t-Tau, total Tau.

^1^ age-corrected *p*-values

^§^ versus PDND

^#^ versus PDD

^$^ versus DLB.

### Cerebrospinal Fluid and Serum Collection

CSF and serum collection was performed according to standardized protocols (for details see [26]). In brief, serum and CSF were centrifuged (blood: 2000 g, 4°C, 10 min; CSF: 4000 g, 4°C, 10 min) and stored at -80°C within 60 minutes after collection until analysis. Only samples with normal routine CSF diagnostics were included (leukocytes < 4x10^6^; IgG index CSF/serum < 0.6, CSF albumin levels < 450 mg/l). Erythrocytes counts in CSF were performed using a semi-quantitative approach (level 1, 0–5 erythrocytes/μL; level 2, >5–1000 erythrocytes/μL; level 3, >1000–5000 erythrocytes/μL). As this is a hypothesis-generating study, GDF15 and MMP9 levels were only analysed in CSF. Serum was used for determination of routine laboratory measures, such as the IgG index.

### Preparation of GDF15 and MMP9 Antibody-Coupled Microspheres

10^6^ microspheres (Luminex cat L100-C104-16 for GDF15 and cat L100-C156-16 for MMP9) were activated for 20 min in 80 μL 1.38% NaH2PO4*H2O, pH 6.0 supplemented with 10 μL sulfo-NHS (Thermo Fisher, Darmstadt, Germany). Activated microspheres were washed with coupling buffer (1% MES, pH 5.0) by briefly vortexing and centrifugation. 200 μL of monoclonal antibody MAB957 (R&D Systems, Minneapolis, USA; 250 μg IgG/mL in 1% MES, pH 5.0) or 200 μL of monoclonal antibody MAB13458 (Millipore, Darmstadt, Germany; 250 μg IgG/mL in 1% MES, pH 5.0) were added to the microspheres and left to react overnight at room temperature in the dark. The coupled microspheres were centrifuged and washed two times with PBS, 0.05% Tween-20. Microspheres were then resuspended in 500 μL PBS, 0.1% BSA, 0.05% proclin300 and stored at 4°C in the dark until use.

### GDF15 Assay

3000 GDF15 antibody coupled beads (diluted into PBS, 0.1% BSA, 0.05% proclin300) were added per well of a prewetted MultiScreen™ HTS filter plate (Millipore). Wells were then drained using a vacuum manifold holder. 25 μL assay buffer (PBS + 0.1% casein + 0.08% CHAPS + 0.25% octylglucopyranoside [OGP]) and either 75 μL of GDF15 standard in assay buffer (recombinant human GDF15; R&D Systems, cat# 957-GD) or CSF diluted in assay buffer were added and incubated overnight at room temperature on a mini orbital shaker. Wells were drained and washed 3 times with 225 μL wash buffer (PBS, 0.05% Tween-20). A mix of 75 μL assay buffer and 25 μL biotinylated goat anti-GDF15 antibody (R&D Systems, cat# BAF940; 5 ng/well, diluted in assay buffer) was then added and incubated for three h at room temperature on a mini orbital shaker. The filter plate was drained and washed three times with 225 μL wash buffer. 100 μL streptavidin R-Phycoerythrin conjugate (SA-PE)(Thermo Fisher, cat# SA1004-1, lot# 495462A; diluted 1/300 in assay buffer) were added and incubated for one h at room temperature on a mini orbital shaker. Filter plates were then drained and washed three times with 225 μL wash buffer. Finally 100 μL PBS was added per well and incubated for 10 min at room temperature on a mini orbital shaker. The median fluorescent intensity (mfi) in region #104 was then determined for each well within one h using the Luminex 100^TM^ IS System (Luminex Corp, Austin, Texas, USA). Each filter plate contained (i) A duplicate GDF15 standard calibrator curve (10000; 3333.33; 1111.11; 370.37; 123.46; 41.15; 13.72; 4.57; 1.52; 0.51 and 0.17 pg/mL) in assay buffer. A stock of frozen aliquots of the different standard dilutions was used for all measurements; (ii) Two serial dilutions (1/15 and 1/60) of each CSF sample that were prepared in assay buffer prior to analysis. Each dilution was measured in duplicate. Results obtained for the 1/15 and 1/60 dilutions showed very high concordance, so only results of the 1/60 dilution experiments are presented. Results of the 1/15 dilution experiments are provided upon request; and (iii) Blank (assay buffer) in duplicate (for further information see S1 File).

### MMP9 Assay

3000 MMP9 antibody coupled beads (diluted in PBS, 0.1% casein) were added per well of a prewetted MultiScreen™ HTS filter plate (Millipore). Wells were then drained using a vacuum manifold holder. 25 μL assay buffer (PBS + 0.1% casein + 0.08% CHAPS + 3% Pluronic F-127) and 75 μL of MMP9 standard in assay buffer (recombinant human MMP9; R&D Systems, cat# 911-MP) or CSF diluted in assay buffer were added and incubated overnight at room temperature on a mini orbital shaker. Wells were drained and washed 3 times with 225 μL wash buffer. A mix of 75 μL assay buffer and 25 μL biotinylated goat anti-MMP9 antibody (R&D Systems, cat# BAF911; 50 ng/well, diluted in assay buffer) was then added and incubated for three h at room temperature on a mini orbital shaker. The filter plate was drained and washed three times with 225 μL wash buffer (PBS, 0.05% Tween-20). 100 μL SA-PE conjugate (Thermo Fisher, cat# SA1004-1, lot# 495462A; diluted 1/300 in assay buffer) were added and incubated for one h at room temperature on a mini orbital shaker. Filter plates were then drained and washed three times with 225 μL wash buffer. Finally 100 μL PBS was added per well and incubated for 10 min at room temperature on a mini orbital shaker. The mfi in region #156 was then determined for each well within one h using the Luminex 100^TM^ IS System. Each plate contained (i) A duplicate MMP9 standard calibrator curve (30,000; 7,500; 1,875; 468.75; 117.19; 29.3; 7.32; 1.83; 0.45; 0.11 and 0.029 pg/mL) in assay buffer. A stock of frozen aliquots of the different standard dilutions was used for all measurements; (ii) Each CSF sample diluted 1/24 in assay buffer and measured in quadruplate; and (iii) Blank (assay buffer) in duplicate (for further information see S1 File).

### Data Processing

Concentrations of the CSF samples were calculated using the StatLIA® v3.2 statistical package (Brendan Technologies Inc., Carlsbad, California, USA) using a 4 (GDF15) or 5 (MMP9) parameter logistic curve with POM (power of mean) weighting.

### Neurodegenerative Marker Analysis

Neurodegenerative markers (CSF Abeta_1-42_, total Tau and phospho-Tau) were determined using Innotest® ELISA kits #80324, #80323, or #80317, respectively (Fujirebio Germany GmbH, Hannover, Germany).

### Data Analysis

Data were analysed with JMP software (Version 11.2.0; SAS Institute Inc., Cary, North Carolina, USA). Demographic and clinical data are presented with median and range / confidence interval (CI). Comparison of GDF15 or MMP9 levels with clinical and routine biochemical parameters were performed using Pearson correlation. Based on results of previous studies (e.g. [27–29]) and on correlations performed in this study indicating at least a weak association of age with GDF15, MMP9, CSF Abeta_1-42_, t-Tau and p-Tau, all calculations including one of these parameters were corrected for age with a logistic regression model. Median GDF15 levels in controls were 16.0% lower for females as compared to males (*p* = 0.03), therefore GDF15 was calculated separately for females and males. We also checked for potentially relevant differences of CSF albumin levels and erythrocytes/μL CSF between controls and patients with a Lewy body disorder, which was not the case (*p* = 0.87 and *p* = 0.70, respectively). As these variables did not show relevant differences between cohorts, we did not include them as covariables in our analyses. Due to the exploratory nature of the study, differences were considered significant at uncorrected *p* < 0.05.

## Results

**Fig 1:**
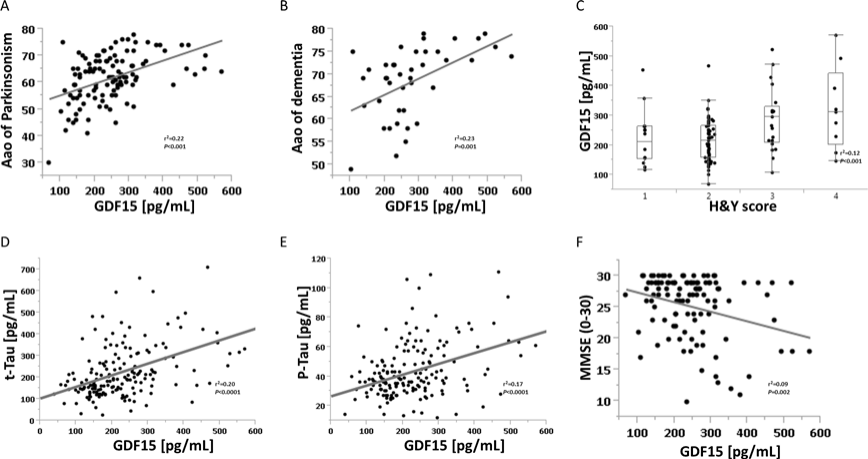
Significant correlations of cerebrospinal fluid GDF15 levels with clinical and routine neurodegenerative markers in Lewy body disorders. (A) GDF15 and age of onset of Parkinsonism. (B) GDF15 and age of onset of dementia. (C) GDF15 and Hoehn and Yahr score. (D) GDF15 and t-Tau. (E) GDF15 and p-Tau. (F) GDF15 and Mini Mental State Examination. Aao, age at onset; CSF, cerebrospinal fluid; GDF15, growth differentiation factor 15; H&Y, Hoehn and Yahr score; MMSE, Mini Mental State Examination; p-Tau, phospho-Tau; r^2^, correlation coefficient; t-Tau, total-Tau.

Patients with a Lewy body disorder had higher CSF GDF15 levels, compared to controls (235 versus 184 pg/mL, *p* < 0.0001). Among patients, age- and gender-corrected median CSF GDF15 levels in the PDD subgroup were 18% higher than in the DLB subgroup (95% CI 6–24%), 16% higher than in the PDND subgroup (6–27%), and 17% higher than the control group (7–28%). In the overall Lewy body disorder cohort, GDF15 was positively correlated with age at onset of Parkinsonism, age at onset of dementia, H&Y stage, and with the neurodegenerative markers t-Tau and p-Tau. CSF GDF15 was negatively associated with the MMSE score (**Fig 1**). It was not significantly associated with any other parameter comprised in the analysis, including disease duration of Parkinsonism (r^2^ = 0.03, *p* = 0.11) and levodopa equivalent daily dose (LEDD, r^2^ = 0.02, *p* = 0.19; Tables 1 and 2).

CSF MMP9 was not significantly associated with any of the included demographic, clinical and routine neurochemical parameters. CSF GDF15 and MMP9 levels did not relevantly correlate. Detailed results structured by included subcohorts are shown in **Table 2**.

**Table 2:** Correlation coefficients (r^2^) of CSF GDF15 and MMP9 and demographic, clinical and neurodegenerative parameters.

			PDND	PDD	DLB	Controls
**GDF15**	**All**	Gender	0.00	0.07	0.29**	0.08**
		Age	0.36***	0.38**	0.15	0.45***
		Aao Parkinsonism	0.29***	0.19	0.16	-
		Duration of Parkinsonism	0.00	0.06	0.00	-
		H&Y stage	0.06	0.38**	0.00	-
		Aao dementia	-	0.30*	0.13	-
		Duration of dementia	-	0.21	0.00	-
		MMSE	0.01	0.26*	0.00	0.01
		LEDD	0.07*	0.18	0.03	-
		CSF Abeta_1-42_	0.04^1^	0.01^1^	0.29^1^*	0.08^1^
		CSF t-Tau	0.49^1^***	0.12^1^	0.30^1^	0.04^1^
		CSF p-Tau	0.43^1^***	0.08^1^	0.19^1^	0.01^1^
	**f**	Age	0.39***	0.61*	0.34*	0.49***
		Aao Parkinsonism	0.40***	0.47	0.30	-
		Duration of Parkinsonism	0.09	0.04	0.01	-
		H&Y stage total	0.03	0.23	0.04	-
		Aao dementia	-	0.51*	0.37*	-
		Duration of dementia	-	0.75**	0.02	-
		MMSE	0.01	0.26	0.23	0.00
		LEDD	0.11	0.34	0.07	-
		CSF Abeta_1-42_	0.17^1^	0.92^1^**	0.10^1^	0.17^1^
		CSF t-Tau	0.68^1^***	0.05^1^	0.40^1^	0.09^1^
		CSF p-Tau	0.74^1^***	0.01^1^	0.19^1^	0.10^1^
	**m**	Age	0.33**	0.32	0.23	0.45***
		Aao Parkinsonism	0.19*	0.12	0.20	-
		Duration of Parkinsonism	0.12	0.08	0.02	-
		H&Y stage total	0.13	0.44	0.01	-
		Aao dementia	-	0.24	0.16	-
		Duration of dementia	-	0.06	0.00	-
		MMSE	0.05	0.20	0.13	0.06
		LEDD	0.04	0.09	0.00	-
		CSF Abeta_1-42_	0.01^1^	0.02^1^	0.48^1^	0.03^1^
		CSF t-Tau	0.35^1^*	0.62^1^	0.41^1^	0.02^1^
		CSF p-Tau	0.20^1^	0.45^1^	0.46^1^	0.00^1^
**MMP9**		Gender	0.00	0.05	0.08	0.01
		Age	0.02	0.01	0.07	0.01
		Aao Parkinsonism	0.00	0.05	0.05	-
		Duration of Parkinsonis	0.01	0.04	0.01	-
		H&Y stage total	0.02	0.00	0.07	-
		Aao dementia	-	0.01	0.05	-
		Duration of dementia	-	0.04	0.02	-
		MMSE	0.00	0.00	0.06	0.00
		LEDD	0.02	0.11	0.00	-
		CSF Abeta_1-42_	0.00^1^	0.00^1^	0.01^1^	0.09^1^
		CSF t-Tau	0.10^1^	0.16^1^	0.21^1^	0.02^1^
		CSF p-Tau	0.13^1^	0.14^1^	0.15^1^	0.01^1^

Pearson correlation and a logistic regression model including age as a covariate ^1^ were used to calculate the extent of correlation between both, growth differentiation factor 15 (GDF15) and matrix metalloproteinase 9 (MMP9), with demographic and clinical parameters of Lewy body disorder subcohorts. The correlation coefficient (r^2^) is presented. Aao, Age at onset; Abeta_1-42_, Amyloid-beta_1-42_; CSF, cerebrospinal fluid; DLB, dementia with Lewy bodies; f, female; H&Y, Hoehn & Yahr stage; LEDD, Levodopa equivalent daily dose; m, male; MMSE, Mini Mental State Examination; PDD, Parkinson`s disease demented; PDND, Parkinson`s disease non-demented; p-Tau, phospho-Tau; t-Tau, total Tau.

* *p* < 0.05

** *p* < 0.01

*** *p* < 0.001.

## Discussion

The results of this study indicate that CSF GDF15 but not MMP9 has potential to (i) differentiate Lewy body disorder patients from controls, (ii) show severity of the disease independent of disease duration, and (iii) even detect prodromal phases of the disorder. Specifically the relatively close associations of GDF15 with t-Tau and p-Tau levels are remarkable. T-Tau is associated with neuronal damage and death [30], and p-Tau with axonal pathology [31]. Both processes [32] as well as alpha-synuclein pathology in general [33,34] have been repeatedly associated with inflammation and remodelling, which makes it suggestive that GDF15 is indeed involved in dopaminergic cell death and Lewy body pathology, not only in animal models (e.g. [3,35–38]) but also in humans.

CSF GDF15 levels were comparably low in controls and PDND patients (who had a relatively short disease duration of 4.5 years, mild motor signs and good cognitive function), intermediate in DLB patients (who had relatively good motor, but poor cognitive functioning), and highest in PDD patients (who had most regularly poor motor *and* poor cognitive functions). Consistent with this observation, disease severity parameters (H&Y and MMSE) were accordingly correlated with GDF15 levels. Hence, comparable to Tau levels, GDF15 levels seem to be directly associated with severity and co-occurrence of symptoms in Lewy body disorders, rather than with specific subtypes and with “simple” disease duration. This observation is, indirectly and outside the Lewy body field, confirmed by studies with initially healthy older adults and patients with brain tumours, which found higher levels of GDF15 associated with increased decline in global cognition, executive function, memory, and processing speed, and increased mortality [10,39].

Although this is a cross-sectional analysis, our results suggest that CSF GDF15 levels have even a potential to detect prodromal stages of Lewy body disorders. In our study, GDF15 levels were higher in control males than in females. If we assume that higher levels indicate more disease “activity”, GDF15 may serve as a surrogate marker for Lewy body disorder even in prodromal disease stages, as risk for Lewy body disorders is higher in males than in females [40]. Interestingly, a recent study [41] observed higher baseline levels of biomarkers indicative of inflammation and remodelling in males compared to females, suggesting higher biological activity in these pathophysiological pathways in males compared to females. Moreover, GDF15 levels were positively and closely correlated with age at onset of Parkinsonism and dementia. In other words, the higher the CSF GDF15 levels, the later occurred the disease itself, and cognitive deterioration within the disease which is closely associated with the disorder.

Taken together, CSF GDF15 levels show surprisingly high association values with relevant clinical, demographic and biochemical parameters of Lewy body disorder, with negative associations with severity of symptoms. We therefore hypothesize that GDF15 as a central member of neuroprotective mechanisms is induced by brain injury [42], such as neurodegeneration. These mechanisms obviously include further anti-inflammatory species and neurotrophic factors. In a recent study using multiplex panels, we observed increased levels of such species and factors (note that we could not determine GDF15 levels due to technical issues) in PD patients compared to controls [43]. Moreover, it may well be that a subtype of PD / DLB exists that is particularly influenced by these anti-inflammatory species and neurotrophic factors: We found that PD patients with a *LRRK2* mutation who displayed a diffuse-malignant phenotype [44], showed higher levels compared to PD *LRRK2* patients with the much more benign pure motor phenotype [45].

Use of MMP inhibitors in Parkinson’s disease might show great promise as the death of dopaminergic neurons seems to be associated with release of MMPs by the activated cells around them [46], making the definition of useful outcome parameters inevitable. However, CSF MMP9 levels between patients with Lewy body disorder and controls did not differ significantly in our analyses, indicating that this parameter does not have a high potential as trait marker for the disease. Moreover, none of the demographic, clinical and neurochemical markers showed significant correlations with CSF MMP9 levels, arguing against the potential of CSF MMP9 as state and predictive marker of the disease. Therefore, even if MMP inhibitors will be tested in Lewy body disorders, the usefulness of at least CSF MMP9 as an outcome parameter remains unclear at present.

The study has limitations. First, we measured a cross-sectional sample, and, particularly as the results indicate that CSF GDF15 is associated with severity of disease, longitudinal information will be necessary to clarify the specific role of the protein with respect to prediction and evaluation of disease severity in Lewy body disorders. Second, due to the complex collection process, cohort sizes are relatively small, and a second study using independent samples is certainly necessary to validate our results. The strength of the study is the examination of biofluid samples from one biobank with highly standardized and fast sample processing and storage procedures, including only material from patients and controls evaluated by movement disorder specialists.

In conclusion, CSF levels of GDF15, but not MMP9, are associated with occurrence and severity in Lewy body disorders. CSF GDF15 may therefore serve as an interesting parameter for, e.g. a panel that aims at differentiating Lewy body disorders from controls, and for progression studies in this arena. Moreover, the direct correlation of CSF GDF15 with age at onset of Parkinsonism and dementia as well as the gender difference of the parameter suggests that GDF15 in CSF has some potential to detect prodromal phases of the disease.

## Supporting Information

S1 File Supplementary material to GDF15 and MMP9 assays. (DOCX)
