# Supplementary material for: GDF15/MIC1 and MMP9 Cerebrospinal Fluid Levels in Parkinson’s Disease and Lewy Body Dementia
Source: PLoS One. 2016 Mar 3;11(3):e0149349. doi: 10.1371/journal.pone.0149349 (PMC4777571; doi:10.1371/journal.pone.0149349)
Supplement: S1 File — (DOCX) [file pone.0149349.s001.docx]

# S1 Supporting information: Supplementary Material to GDF15 and MMP9 assays

# GDF15 assay

## **Analytical validation of the GDF15 assay**.

We determined the total error (measurement error) of the GDF15 analytical assay in a series of validation experiments [REF (1)]. The total error is composed of the trueness (bias) and the precision. It is not trivial to determine the bias of the procedure as a gold standard for GDF15 does not exist, CSF always contains endogenous GDF15 and would have to be depleted of GDF15 before spiking of the standard and reactivity of endogenous GDF15 with the antibodies might differ from that of the standard GDF15. We therefore assumed that the bias was constant and arbitrarily set it at zero.

For the validation experiments two kinds of samples were prepared: calibration standards and validation samples. GDF15 calibration standards were made by serial 3-fold dilution of a GDF15 stock solution in assay buffer (PBS + 0.1% casein + 0.08% CHAPS + 0.25% OGP; 1000, 333.33, 111.11, 37.04, 12.34, 4.11, 1.37, 0.46, 0.15 and 0.051 pg/mL). The validation samples were prepared by 3-fold serial dilution (1/8, 1/24 and 1/72) of 5 different human CSF samples (BOCA-0210A284, BOCA-0210A286, BOCA-0210A289, BOCA-0210A305 and BRH403517) in assay buffer. Each dilution of the standard and CSF samples was split into many small aliquots which were stored at -70°C. After thawing, aliquots were immediately used as calibration or validation samples in the experiment. Excess thawed liquid was discarded.

For the validation experiments the GDF15 calibration standards and CSF validation samples were measured in duplicate in 6 independent experiments (on 6 different days) by the same operator. In each run, GDF15 calibration standards were measured and used to determine the GDF15 levels in the different validation samples.

## Calibration curves of the GDF15 assay.

## The standard dilution curves of the 6 batch runs displayed limited variability (Fig 1). Concentration-response curves were established using different regression models with or without weighting (Seelva V1.0 beta 14 software; Arlenda, Saint-Georges, Belgium). Different models were sorted according to a composite index incorporating bias, precision and analytical range. A four parameter weighted (POM: power of mean) logistic regression model (4PL) was found to generate the best accuracy profile (Fig 1).


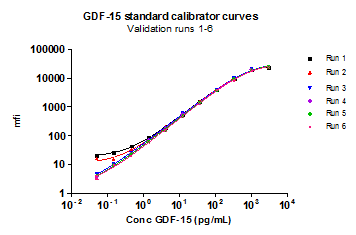


Fig 1. *Calibration curves of the GDF15 Luminex assay: validation runs. Weighted 4PL fitted concentration-response curves of the GDF15 calibrator were determined in 6 independent runs. Dilutions of recombinant GDF15 were made in assay buffer and measured in duplicate.*

## Assay linearity: parallelism in human CSF of the GDF15 assay.

Assay linearity depends on the parallelism between the calibrator curve and the dilution response curves of CSF. We assessed the parallelism of the GDF15 assay in human CSF as follows. 5 human CSF samples (BOCA-0210A290, BOCA-0210A301, BRH403514, BRH403515 and BRH403518) were measured in 6 independent runs (on 6 independent days). Each CSF sample was measured in duplicate at 3 serial dilutions (1/8, 1/24 and 1/72) in the assay buffer. In each run a duplicate standard curve was also included. Concentration-response curves were generated by POM weighted 4PL curve fitting as described above.

We investigated the degree of parallelism of the CSF dilution-response curves to the GDF15 standard calibrator curves, by calculating the coefficient of variation (CV) of the dilution-adjusted concentrations determined at different dilutions of each CSF sample (between-dilution CV). The measured dilution-adjusted concentrations were plotted against the dilution factor (Fig 2).

Dilution-adjusted concentrations did not show a monotonous increase or decrease in function of the dilution and showed between-dilution CVs below 15% for all the CSF samples in the 8x - 72x dilution range and even below 10% in most of those. These characteristics comply with literature recommendations [REF (2)].

The above experiment thus demonstrated that the GDF15 Luminex assay exhibits parallelism between CSF dilution curves and the GDF15 standard curve in assay buffer in the 8x - 72x dilution range. This demonstrates the linearity of the assay and indicates that the human CSF matrix does not interfere with the GFD15 assay.


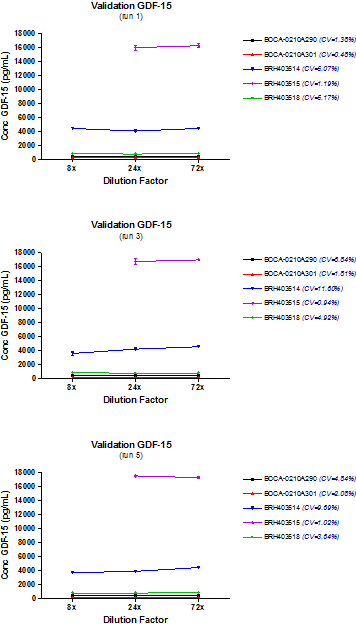


Fig 2. *Validation of the GDF15 Luminex assay: Determination of parallelism between the calibrator curve in assay buffer and dilution curves of human CSF samples (all 5 from normal donors). In total 6 independent runs were done (only runs 1-3-5 are shown here). Concentrations were calculated based on the standard curve in assay buffer. Dilution-adjusted concentrations are plotted against the dilution factor. The between-dilution CVs are indicated between brackets.*

## Accuracy Profile of the GDF15 assay.

## Fig 3 shows the accuracy profile (total relative error) of the MAB957 / BAF940 GDF15 Luminex assay in human CSF as estimated from the validation experiments using the Seelva V1.0 beta 14 software (Arlenda, Saint-Georges, Belgium). As the bias was assumed to be unknown but constant, it was arbitrarily set at 0%. The relative error thus reflects the precision of the assay. The acceptance criteria were set at 20%. The method was considered valid within the range for which the 80% expectation interval was within the accuracy acceptance limits. The analytical range for the GDF15 assay was found to be 2.36 - 368.4 pg/mL (lower limit of quantification [LLOQ] - upper limit of quantification [ULOQ]).


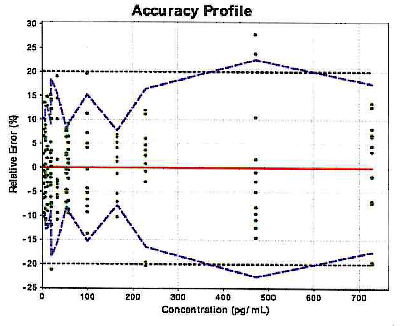


Fig 3. *Accuracy profile of the GDF15 assay in CSF. Since bias (solid red line) was arbitrarily set at 0%, the percentage relative error actually reflects precision. 80% expectation interval (dashed blue lines) is plotted for different GDF15 concentrations. The dotted lines represent the acceptance limits (set at 20% relative error).*

## Absence of cross-reactivity of the GDF15 assay.

Based on the very low degree of sequence similarity between the mature GDF15 protein and other members of the TGF-beta family it was judged that the possibility for cross-reactivity was extremely low (especially for a sandwich ELISA as both antibodies would need to cross-react). The capture monoclonal antibody MAB957 was commercially obtained (R&D Systems Inc., Minneapolis, USA). The data sheet of this antibody specifies that in ELISA the antibody does not cross-react with recombinant human GDF-11, recombinant mouse (rm) GDF-5, rmGDF-6, rmGDF-7 or rmGDF-8. Protein sequence alignment of human GDF15, human GDF-11, human TGF-beta1, human TGF-beta2 and human TGF-beta-3 illustrates the low degree of conservation between these proteins, with the GDF-11 being more similar to TGF-beta 1/2/3 than to GDF15 (Fig 7). Given the absence of cross-reactivity of MAB957 with GDF-11 it appears highly unlikely that it will cross-react with TGF-beta.

# Analytical validation of the MMP9 assay.

For the validation experiments two kinds of samples were prepared: calibration standards and validation samples. MMP9 calibration standards were made by serial 4-fold dilution from a MMP9 stock in assay buffer (PBS + 0.1% casein + 0.08% CHAPS + 3% PF-127; 30000, 7500, 1875, 468.75, 117.19, 29.30, 7.32, 1.83, 0.46, 0.11 and 0.029 pg/mL). The validation samples were prepared by 3-fold serial dilution (1/8, 1/24 and 1/72) of 5 different human CSF samples (BOCA-0210A281, BOCA-0210A294, BOCA-0210A296, BOCA-0210A306 and BRH403515) in assay buffer. Each dilution of the standard and CSF samples was split into many small aliquots which were stored at -70 °C. After thawing, aliquots were immediately used as calibration or validation samples in the experiment. Excess thawed liquid was discarded.

For the validation experiments, the MMP9 calibration standards and CSF validation samples were measured in quadruplicate in 6 independent experiments (on 6 different days) by the same operator. In each run, MMP9 calibration samples were measured and used to determine the MMP9 levels in the different validation samples.

## **Calibration curves of the MMP9 assay**.

The standard dilution curves of the 6 batch runs displayed limited variability (Fig 4). Concentration-response curves were established using different regression models with or without weighting (Seelva V1.0 beta 14 software; Arlenda, Saint-Georges, Belgium). Different models were sorted according to a composite index incorporating bias, precision and analytical range. A five parameter weighted (POM: power of mean) logistic regression model (5PL) was found to generate the best accuracy profile.

Fig 4. *Calibration curves of the MMP9 Luminex assay: validation runs. Weighted 5PL fitted concentration-response curves of the MMP9 calibrator were determined in 6 independent runs. Dilutions of recombinant MMP9 were made in assay buffer and measured in quadruplicate.*

## Assay linearity: parallelism in human CSF of the MMP9 assay.

We assessed the parallelism of the MMP9 assay in human CSF as follows. 5 human CSF samples (BOCA-0210A277, BOCA-0210A278, BOCA-0210A288, BOCA-0210A302 and BOCA-0210A304) were measured in 6 independent experiments (on 6 independent days). Each CSF sample was measured in quadruplicate at serial dilutions (1/8, 1/24 and 1/72) in the assay buffer. In each experiment a quadruplicate calibrator dilution series was also included. Calibrator curves were generated by a weighted 5PL curve fitting as described above.

We investigated the degree of parallelism of the CSF dilution-response curves to a MMP9 standard calibrator curve, by calculating the CV of the dilution-adjusted concentrations determined at different dilutions of each CSF sample (between-dilution CV). The measured dilution-adjusted concentrations were plotted against the dilution factor (Fig 5).

Dilution-adjusted concentrations did not show a monotonous increase or decrease in function of the dilution and showed between-dilution CVs of below 20% for all the CSF samples in the 8x - 72x dilution range and even below 10% in most of those. These characteristics comply with literature recommendations [REF (2)].

The above experiment thus demonstrated that the MMP9 Luminex assay exhibits parallelism between CSF dilution curves and the MMP9 standard curve in assay buffer in the 8x - 72x dilution range. This indicates that human CSF matrix does not interfere with the MMP9 assay.


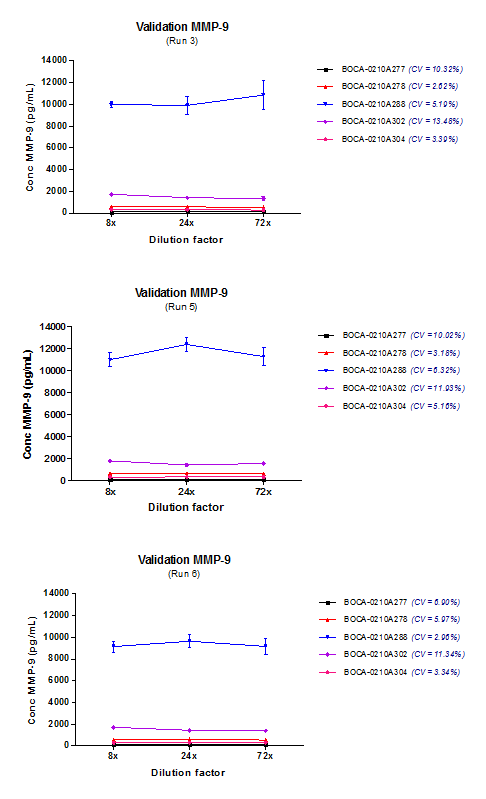


Fig 5. *Validation of the MMP9 Luminex assay: Determination of parallelism between the calibrator curve in assay buffer and dilution curves of human CSF samples (all 5 from normal donors). In total 6 independent runs were done (only runs 3-5-6 are shown here). Concentrations were calculated based on the standard curve in assay buffer. Dilution-adjusted concentrations are plotted against the dilution factor. The between-dilution CVs are indicated between brackets.*

## Accuracy Profile of the MMP9 assay.

Fig 6 shows the accuracy profile (total relative error) of the MAB13458 / BAF911 MMP9 Luminex assay in human CSF as estimated from the validation experiments using the Seelva v1.0 beta14 software (Arlenda, Saint-Georges, Belgium). As the bias was assumed to be constant but unknown it was arbitrarily set at 0%. The relative error thus reflects the precision of the assay. The acceptance criteria were set at 30%. The method is considered valid within the range for which the 80% expectation interval is within the accuracy acceptance limits. The analytical range is thus 1.83 – 1258 pg/mL (LLOQ-ULOQ).


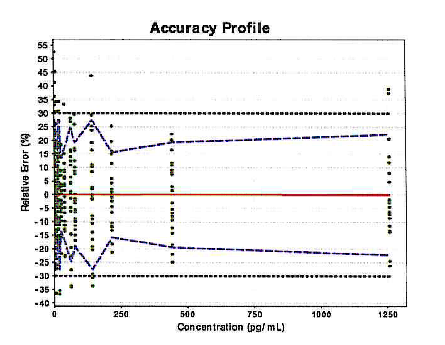


Fig 6. *Accuracy profile of the MMP9 assay in CSF. Bias (solid red line, arbitrarily set at 0%) and 80% expectation interval (dashed blue lines) are plotted for different MMP9 concentrations. The dotted lines represent the acceptance limits (30%).*

**
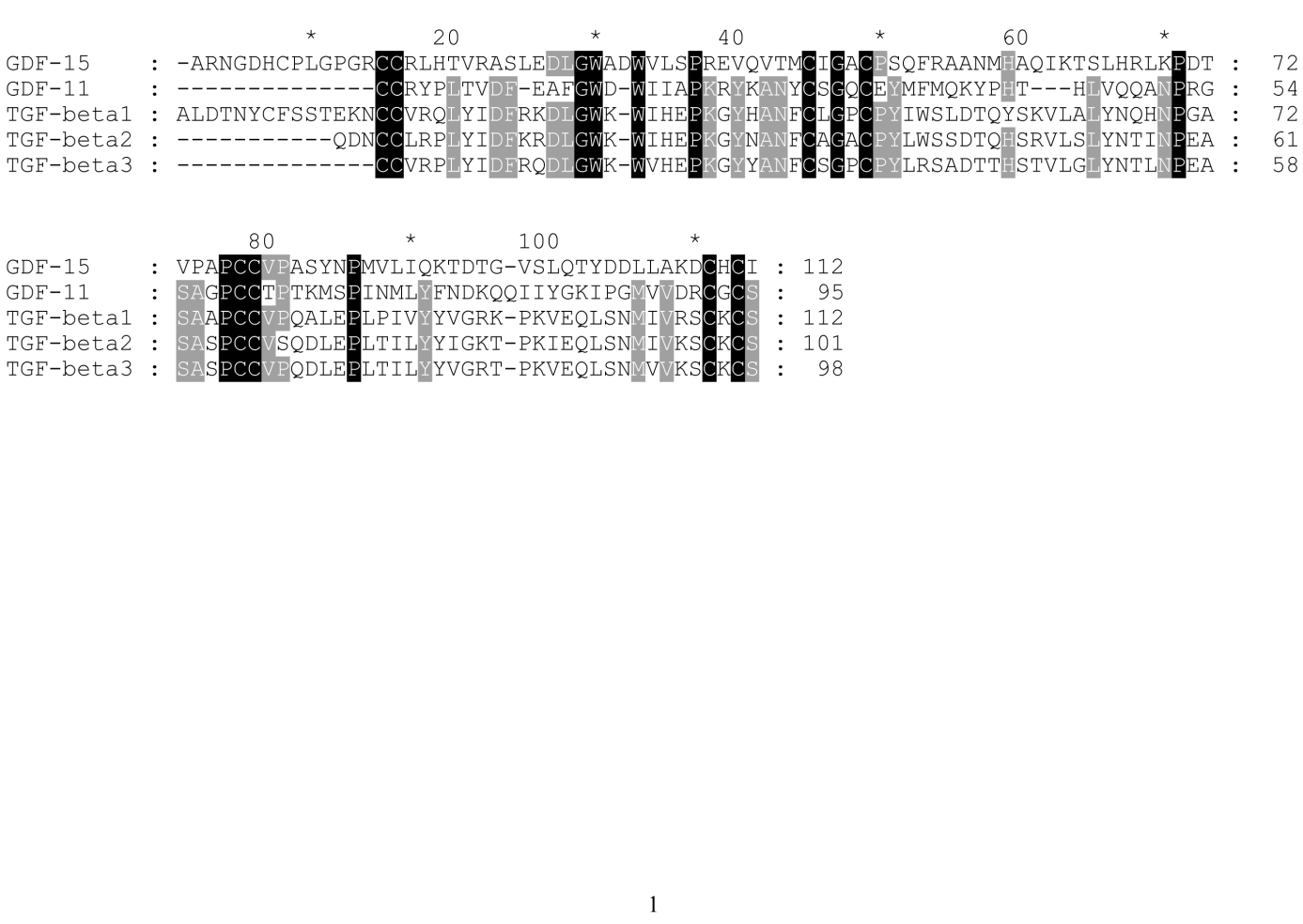
**

Fig 7. Sequence alignment of mature human GDF15, GDF-11, TGF-beta1, TGF-beta2 and TGF-beta3.

# References

1. Boulanger B, Devanarayan V, Dewé W. Statistical considerations in the validation of ligand-binding assays. In: Khan MN, Findlay JWA, editors. Ligand-Binding Assays: Development, Validation, and Implementation in the Drug Development Arena. Wiley & Sons, Inc.; 2010.

2. Plikaytis BD, Holder PF, Pais LB, Maslanka SE, Gheesling LL, Carlone GM. Determination of parallelism and nonparallelism in bioassay dilution curves. Journal of clinical microbiology [Internet]. 1994 Oct [cited 2015 Dec 11];32(10):2441–7.
